# Sub-Classification of Cirrhosis Affects Surgical Outcomes for Early Hepatocellular Carcinoma Independent of Portal Hypertension

**DOI:** 10.3389/fonc.2021.671313

**Published:** 2021-05-20

**Authors:** Er-lei Zhang, Jiang Li, Jian Li, Wen-qiang Wang, Jin Gu, Zhi-yong Huang

**Affiliations:** Hepatic Surgery Center, Tongji Hospital, Tongji Medical College, Huazhong University of Science and Technology, Wuhan, China

**Keywords:** cirrhosis, histological, hepatocellular carcinoma, Laennec staging, portal hypertension, liver resection

## Abstract

Severity of liver cirrhosis is distinct from clinical portal hypertension because there exist different degrees of liver cirrhosis in hepatocellular carcinoma (HCC) patients without significant clinical portal hypertension. Whether severity of cirrhosis affects surgical outcomes for HCC patients in absence of portal hypertension or not remains unclear. This study aims to analyze the effect of cirrhotic severity on surgical outcomes for HCC patients with hepatitis B virus (HBV) infection in absence of portal hypertension. This retrospective study enrolled 166 patients who underwent curative resection for a single HCC ≤5 cm in absence of portal hypertension between February 2011 and December 2013. Liver cirrhosis was sub-classified into no/mild (no/F4A) and moderate/severe (F4B/F4C) according to the Laennec scoring system. The surgical outcomes and complications were analyzed. The surgical mortality was zero in this study. Major complications were apparently higher in the F4B/F4C group than in the no/F4A group (17.0% vs 7.4%, *p <*0.001). The 1-year, 3-year and 5-year overall survival (OS) rates were 98.5, 88.1 and 80%, respectively, in the no/F4A group, which were significantly higher than those in the F4B/F4C group (98.0, 69.2 and 54.7%, *p* = 0.001). Microscopic vascular invasion, absence of tumor capsule and severity of liver cirrhosis were independent risk factors of surgical outcomes for HCC patients without portal hypertension. In conclusion, severity of liver cirrhosis affected surgical outcomes for early-stage HCC patients independent of portal hypertension.

## Introduction

Hepatocellular carcinoma (HCC) is the fifth most common cancer worldwide and the second most common cause of cancer mortality (1). In China, more than four fifths of HCC patients have presented with varied degrees of liver cirrhosis (2). For the past ten years, due to active surveillance programs, the detection of early-stage HCC has increased, therefore, the number of HCC patients who are suitable for curative treatment has consequently increased. Together with local ablation (LA) and liver transplantation (LT), liver resection (LR) is considered as the first-line treatment for small HCC patients with relatively good liver function, which offers a chance of cure and long-term surgical outcomes. However, the factors affecting surgical outcomes for early-stage HCC concomitant with liver cirrhosis remain a major concern. It should be noted that the prognosis of HCC patients was influenced not only by the tumor status but also underlying liver cirrhosis. In addition, tumor recurrence remains a major issue in clinical management of HCC and the cumulative risk of recurrence during the first five years after LR is still high (3, 4). The high risk of recurrence after curative-intent LR is attributable to the following two patterns: recurrence derived from residual micro-metastases and *de novo* recurrence due to the underlying liver cirrhosis (5, 6). Tumor recurrence is associated with the different degrees of underlying liver cirrhosis (7) and the annual incidence of HCC in patients with liver cirrhosis has been reported to range between 2.5 and 6.6% (8–10). The cumulative incidence of recurrence owing to *de novo* carcinogenesis would result in significant differences in surgical outcomes between patients with liver cirrhosis versus those with normal liver (11, 12). Moreover, liver cirrhosis is a dynamic process and the severity and clinical prognosis vary greatly, even among HCC patients within the same histological degree of cirrhosis (10, 13, 14). It needs to further reveal the impact of histological sub-classification of cirrhosis on surgical outcomes after LR for HCC patients.

The Laennec scoring system histologically subdivided liver cirrhosis into three stages (F4A, F4B and F4C) according to the thickness of the fibrous septa and size of the nodules (15). Studies reported that histological sub-classification of liver cirrhosis using the Laennec scoring system could predict late recurrence in HCC patients after curative resection, and it was obviously correlated with grade of portal hypertension but not the same disease (15, 16). However, there exist an obvious heterogeneity within cirrhosis even in HCC patients without significant portal hypertension, and the importance of cirrhotic severity in clinical practice should be further validated in this circumstance.

More and more surgeons have realized the key role of cirrhotic severity in surgical modalities and long-term outcomes (10, 16–18). The present study aimed to elucidate whether histological sub-classification of liver cirrhosis affected surgical outcomes of the early-stage HCC patients in absence of significant portal hypertension.

## Materials and Methods

### Patients

From February 2011 to December 2013, 1,187 patients underwent liver resection (LR) for the first time in the Hepatic Surgery Center at Tongji Hospital of Tongji Medical College, Huazhong University of Science and Technology. HCC was diagnosed based on cyto-histological evidence from excised specimens. Demographic characteristics, laboratory parameters, imageological and histological results of all the selected patients were all collected. Portal hypertension was indirectly diagnosed according to the following criteria: esophageal varices by endoscopy or total platelet count <100,000/ml combined with splenomegaly (19). A total of 166 patients met the following inclusion criteria:

1. Early-stage HCC (solitary lesions less than 5 cm) with HBV infection.
2. No portal vein tumor thrombus or extra-hepatic metastases.
3. Child–Pugh A liver functions and absence of portal hypertension.
4. No previous treatments for HCC.
5. Patients with no severe comorbidities that cannot tolerate surgery.

**Figure S1** shows a flow diagram of enrolled HCC patients in the present study.

### Surgical Treatment

LR was carried out in patients who satisfied the surgical indication with central venous pressure (CVP) less than 5mmHg using a right sub-costal incision. Intra-operative ultrasound was routinely performed to confirm tumor location and satellite nodules, as well as assess the vascular anatomy of the liver. We performed all the hepatectomies with R0 resection. The Cavitron Ultrasonic Aspiration (CUSA, Valleylab Corp, USA) and Harmonic scalpel (Johnson & Johnson Ltd, USA) were used to transect liver parenchyma. Pringle’s maneuver was performed intermittently, each time for less than 15 min, with an interval of 5 min aiming to minimize peri-operative blood loss.

### Histological Evaluation of Liver Specimens

Histological evaluation of liver specimens was carried out by two experienced pathologists blinded to clinical information according to the Laennec scoring system (15). The samples were re-examined to analyze for discrepancies and a consensus was reached when the two pathologists get the inconsistent results. Liver cirrhosis was evaluated in non-cancerous tissues and was scored on four scales depending on the Laennec scoring system: F0–F3: no cirrhosis; F4A: mild cirrhosis (most septa are thin, allowing only one broad septum); F4B: moderate cirrhosis (at least two broad septa); F4C: severe cirrhosis (more than one very broad septum or many micro-nodules). According to the comparison between the thickness of fibrous septa and the nodule size, “broad septum” was defined as septal thickness being thinner than nodule size, and when the septal thickness is thicker than nodule size, “very broad septum” was diagnosed (15, 20).

### Follow-Up and Efficacy

For HCC patients with chronic HBV infection, Adefovir Dipivoxil 10 mg or Entecavir 0.5 mg was orally administered daily when the pre-operative HBV DNA was positive. All patients were continuously followed up follow up every 2 months during the first 6 months after operation or 3 months thereafter. Surveillance for HCC included serum alpha-fetoprotein (AFP) level, chest radiography and abdominal ultrasonographic examination. Postoperative enhanced Computed Tomography (CT) or Magnetic Resonance Imaging (MRI) scans were performed every three months if necessary. HCC recurrence was diagnosed on the basis of the two consistent imaging examinations or the combination of increased AFP and one imaging result with consistent radiologic features of HCC. LR was performed when recurrence occurred if it was suitable for surgery according to the same criteria with the first surgical resection. If the patients were not suitable for LR, transarterial chemoembolization (TACE), local ablation or systemic therapy were applied. The overall survival (OS) was calculated from the day of operation to final follow-up or death. The disease-free survival (DFS) was calculated from the date of operation to the date when recurrence/metastasis was diagnosed.

### Statistical Analysis

Clinico-pathological parameters were expressed as frequencies and percentages for qualitative variables and mean ± SD or median (range) for continuous variables. Significance of differences between the groups were evaluated using Student’s t-test. Descriptive variables were analyzed with χ2 or Fisher’s exact test as appropriate. Surgical outcomes were estimated using the Kaplan–Meier curve and the log-rank test was used to compare the survival rates among all the groups. All significant predictors of OS and DFS in univariate and multivariate analysis were analyzed in cox proportional hazards regression model. All the risk factors that were significant (*p <*0.05) for the prediction of long-term outcomes in an univariate analysis were selected for further multivariate analysis. The 95% confidence intervals (95% CIs) and hazard ratios (HRs) were calculated. All tests were two-tailed and 0.05 was intended to be statistically significant. Statistical analysis was performed using SPSS19.0 (SPSS Inc., Chicago, IL, USA).

## Results

### Patients

A total of 166 patients were enrolled in the present study according to the including criteria. Baseline characteristics of all the included HCC patients were described in **Table 1**. Based on the Laennec scoring system, the proportion of no cirrhosis, mild cirrhosis (F4A), moderate cirrhosis (F4B) and severe cirrhosis (F4C) were 13.2% (n = 22), 27.7% (n = 46), 39.2% (n = 65) and 19.9% (n = 33), respectively. The proportion of patients with microvascular invasion and poor histological grades were 10.8 and 22.9%, respectively. One hundred and twelve (67.5%) tumors had integrated capsule.

**Table 1:** Clinical characteristics and demographics of 166 patients with a solitary hepatocellular carcinoma.

Variables	Value
Age (years)	49.0 ± 10.5 (22–76)
Sex (Male : Female)	148 (89.2): 18 (10.8)
HBV-DNA (IU/L)	
>2,000	70 (42.2%)
≤2,000	96 (57.8%)
ALT (U/L)	37.1 ± 18.4 (9–99)
AST (U/L)	33.0 ± 13.5 (12–89)
Total bilirubin (umol/L)	13 ± 3.8
INR	1.1 ± 0.1
Albumin (g/L)	40.6 ± 4.0
Platelet count (×10^9^/L)	126.6 ± 51.8
AFP (ng/ml)	
>400 (n, %)	52 (31.3)
≤400 (n, %)	114 (68.7)
ICGR-15 (%)	5.2 ± 3.6
Spleen thickness (cm)	3.9 ± 0.7
Tumor size (cm)	3.9 ± 0.9
>3 (n, %)	105 (63.3)
≤3 (n, %)	61 (36.7)
Anatomic resection (n, %)	36 (21.7)
Capsule (n, %)	112 (67.5)
Tumor differentiation (n, %)	
Well	40 (24.1)
Moderate	88 (53.0)
Poor	38 (22.9)
Microscopic vascular invasion (n,%)	
Yes	18 (10.8)
No	148 (89.2)
Laennec fibrosis stage (n, %)	
F0–F3	22 (13.2)
F4A	46 (27.7)
F4B	65 (39.2)
F4C	33 (19.9)

### Mortality and Complications

There was no surgical-related mortality in this study. Major complications occurred in 12% of the patients (n = 20). Depending on the histological severity of liver cirrhosis, we divided all the patients into two groups: no/F4A group (n = 68) and F4B/4C group (n = 98). The surgical complications between the two groups were compared. Major complications were apparently higher in the F4B/F4C group (n = 15) than the no/F4A group (n = 5) (15.3% vs 7.4%, *p <*0.001). Surgical complications and perioperative details of patients in both groups shows in **Table S1**. All the above complications were recovered with conservative therapy while in hospital.

### Recurrence and Treatment

HCC recurrence was found in 88 patients during the follow-up period. The 1-, 3-, 5-year recurrence rates were 11.4, 34.9, 50.6%, respectively. We further subdivided the patients into the no/F4A group (n = 68) and the F4B/4C group (n = 98) according to the Laennec scoring system. Clinico-pathological characteristics showed no significant difference between the two groups except from types of resection (as shown in **Table S2**). Subgroup analysis suggested that the recurrence rates of HCC patients in the F4B/4C group were significantly higher than those in the no/F4A group, the corresponding 1-, 3-, 5-year recurrence rates were 14.3, 42.9, 60.2% versus 7.4, 23.5, 36.8%, respectively (*p <*0.001). Late-stage recurrence rates (>2 years) were still significantly higher in the F4B/4C group compared with those in the no/F4A group (65/98 vs16/68, *p <*0.001).

For the 88 patients with tumor recurrences, 38 patients were treated with Percutaneous Microwave Coagulation (PMCT); 10 patients performed with the second LR; 24 patients received trans-arterial chemotherapy and embolization (TACE); 10 patients were treated with the combination therapy of PMCT and TACE; six patients received TACE combined with systemic therapy (summarized in **Table S3**).

### Survival

**Figure 1:**
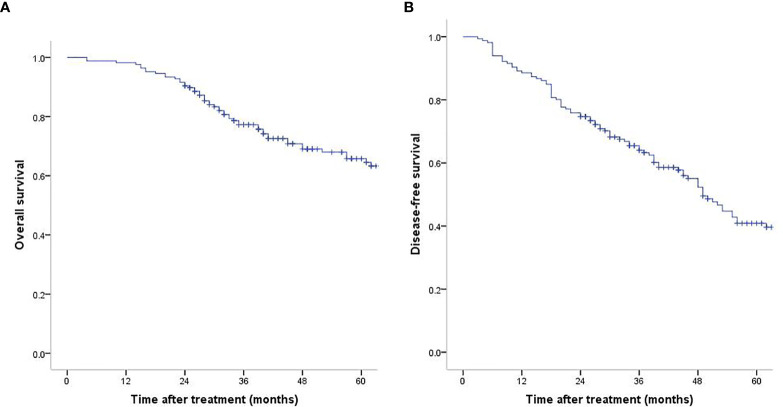
Overall survival **(A)** and disease-free survival **(B)** in the whole study population who underwent liver resection.

**Figure 2:**
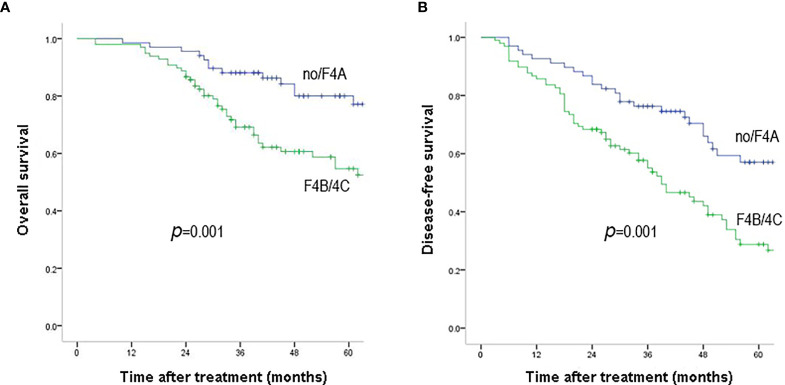
Overall survival **(A)** and disease-free survival **(B)** outcomes of HCC patients with F4B/F4C and no/F4A. Overall survival and disease-free survival rates were significantly better in patients with no/F4A than those with F4B/4C (*P* = 0.001).

During a median of 47.7 months (interquartile range: 16.7–83.6 months) of follow-up, 58 patients (34.9%) were dead. The 1-, 3-, 5-year OS rates were 98.2, 77.2, 65.8% and the corresponding DFS rates were 88.6, 64.0, 40.9% (**Figures 1A, B**). According to the Laennec scoring system, liver cirrhosis was histologically sub-classified into four groups: no cirrhosis (n = 22), mild cirrhosis (F4A, n = 46), moderate cirrhosis (F4B, n = 65), severe cirrhosis (F4C, n = 33). Subgroup analysis indicated that the OS and DFS rates differed significantly among the four groups (**Tables 2** and **S4**). Surgical outcomes decreased significantly with the increasing degrees of liver cirrhosis (as shown in **Figures S2A, B**, *p <*0.001). We further found that the 1-, 3-, 5-year OS rates were 98.5, 88.1, 80% for patients in the no/F4A group and 98.0, 69.2, 54.7% for patients in the F4B/4C group, respectively (**Figure 2A**, *p* = 0.001). The corresponding DFS rates for the two groups were 92.6, 76.3, 57.1% and 85.7, 55.0, 28.8%, respectively (**Figure 2B**, *p* = 0.001).

**Table 2:** Comparison of the overall and disease-free survival among the HCC patients with different degrees of liver cirrhosis.

	1-year (%)	3-year (%)	5-year (%)	*P-*value
Overall survival				
F0–F3 vs.F4A–4C (22/144)	100/97.9	90.2/75.3	82.7/63.2	0.044
F0–F4A vs.F4B–4C (68/98)	98.5/98.0	88.1/69.2	80.0/54.7	0.001
F0–F4B vs.F4C (133/33)	98.5/97	81.2/61.1	72.1/38.9	0.02
F0–F3 vs.F4A (22/46)	100/97.8	90.2/87.0	82.7/78.7	0.381
F4A vs.F4B (46/65)	97.8/98.5	87.0/73.4	78.7/63.0	0.081
F4B vs.F4C (65/33)	98.5/97	73.4/61.6	63.0/38.9	0.09
Disease-free survival				
F0–F3 vs.F4A–4C (22/144)	95.5/87.5	81.6/61.4	59.8/38.1	0.051
F0–F4A vs.F4B–4C (68/98)	92.6/85.7	76.3/55.0	57.1/28.8	0.001
F0–F4B vs.F4C (133/33)	91.7/75.8	70.7/36.5	46.1/18.9	<0.001
F0–F3 vs.F4A (22/46)	95.5/91.3	81.6/73.9	59.8/55.9	0.532
F4A vs.F4B (46/65)	91.3/90.8	73.9/64.5	55.9/33.6	0.082
F4B vs.F4C (65/33)	90.8/75.8	64.5/36.5	33.6/18.9	0.018

Univariate and multivariate cox regression analyses identified that no microscopic vascular invasion (MVI), no capsule and F4B/4C cirrhosis were independent risk factors of long-term outcomes (OS and DFS), as shown in **Tables 3** and **4**.

**Table 3:** Univariate and multivariate analysis of the relative risk of overall survival.

Variables	OS				
	Comparison	Univariate HR (95% CI)	*p*-value	MultivariateHR (95% CI)	*P*-value
Age (years)	>50 vs ≤50	0.857 (0.508,1.447)	0.564		
Sex	Male vs Female	0.972 (0.417, 2.266)	0.947		
HBV-DNA (IU/L)	>2,000 vs ≤2,000	1.001 (0.591,1.697)	0.996		
ALT (U/L)	>35 vs ≤35	1.579 (0.941,2.651)	0.084		
AST (U/L)	>35 vs ≤35	1.444 (0.850,2.453)	0.174		
Total bilirubin (umol/L))	>17 vs ≤17	0.980 (0.493,1.947)	0.955		
INR	>1.2 vs ≤1.2	1.884 (0.891,3.983)	0.097		
Albumin (g/L)	>35 vs ≤35	0.708 (0.303,1.652)	0.424		
Platelet count (×109/L)	>100 vs ≤100	0.779 (0.449,1.352)	0.374		
AFP (ng/ml)	>400vs ≤400	1.595 (0.941,2.703)	0.083		
ICGR-15 (%)	>10 vs ≤10	0.920 (0.332,2.546)	0.872		
Spleen thickness (cm)	>4 vs ≤4	1.310 (0.776,2.212)	0.311		
Tumor size (cm)	>3 vs ≤3	1.153 (0.670,1.982)	0.607		
Anatomic resection	Yes vs No	0.899 (0.484,1.671)	0.737		
MVI	Yes vs No	3.003 (1.664,5.417)	<0.001	2.652 (1.298,5.419)	0.007
Capsule	Yes vs No	0.447 (0.264,0.758)	0.003	0.419 (0.245,0.715)	0.001
Tumor differentiation	Poor vs well and moderate	2.214 (1.046,4.688)	0.038	1.973 (0.891,4.367)	0.094
Laennec fibrosis stage	Moderate/severe vs no/mild	2.256 (1.417,4.626)	0.002	2.145 (1.156,3.980)	0.016

**Table 4:** Univariate and multivariate analysis of the relative risk of disease-free survival.

Variables	DFS				
	Comparison	Univariate HR (95% CI)	*p*-value	MultivariateHR (95% CI)	*p*-value
Age (years)	>50 vs ≤50	0.923 (0.606, 1.406)	0.709		
Sex	Male vs Female	0.706 (0.374, 1.330)	0.281		
HBV-DNA (IU/L)	>2,000 vs ≤2,000	1.378 (0.904,2.101)	0.136		
ALT (U/L)	>35 vs ≤35	1.064 (0.699,1.619)	0.771		
AST (U/L))	>35 vs ≤35	1.152 (0.743,1.785)	0.527		
Total bilirubin (umol/L))	>17 vs ≤17	0.944 (0.539,1.652)	0.839		
INR	>1.2 vs ≤1.2	1.577 (0.789,3.155)	0.198		
Albumin (g/L)	>35 vs ≤35	0.934 (0.450,1.935)	0.853		
Platelet count (×109/L)	>100 vs ≤100	0.826 (0.526,1.296)	0.406		
AFP (ng/ml)	>400vs ≤400	1.558 (1.007,2.410)	0.046	1.492 (0.942, 2.364)	0.088
ICGR-15 (%)	>10 vs ≤10	1.421 (0.685,2.946)	0.345		
Spleen thickness (cm)	>4 vs ≤4	1.421 (0.930,2.171)	0.104		
Tumor size (cm)	>3 vs ≤3	1.552 (0.980,2.455)	0.060		
Anatomic resection	Yes vs No	0.845 (0.508,1.404)	0.515		
MVI	Yes vs No	3.003 (1.664,5.417)	<0.001	3.163 (1.658,6.032)	<0.001
Capsule	Yes vs No	0.466 (0.302,0.717)	0.001	0.387 (0.248,0.603)	<0.001
Tumor differentiation	Poor vs well/moderate	2.223 (1.204,4.104)	0.011	1.915 (0.991,3.699)	0.053
Laennec fibrosis stage	Moderate/severe vs no/mild	2.130 (1.351,3.358)	0.001	1.928 (1.187,3.134)	0.008

## Discussion

Most histological scoring systems of fibrosis regarded cirrhosis as the end stage. In the past few years, increasing evidence indicated that there existed an obvious histological difference within cirrhosis, and liver cirrhosis should be further sub-classified based on its histological severity (10, 14, 15, 17, 18, 20). The BCLC guideline advocated that LR was recommended only for HCC patients without clinical portal hypertension and with normal total bilirubin levels. Histological sub-classification of liver cirrhosis is tightly correlated with the grade of clinical portal hypertension but not the same disease (15). For those HCC patients without portal hypertension, the importance of the underlying severity of liver cirrhosis should be further emphasized. Actually, most of HCC patients arise from varied degrees of liver cirrhosis in China (2). It is essential to enlighten on the value of histological sub-classification of cirrhosis in predicting potential surgical outcomes of HCC and individualize therapy. Till date, the role of the severity of liver cirrhosis in affecting the surgical outcomes for HCC patients without significant portal hypertension remains unclear. In this study, we aimed to analyze the effect of cirrhotic severity on surgical outcomes for HBV-related HCC patients in absence of portal hypertension.

Current guidelines recommended LR as the first-line treatment for a single HCC with Child–Pugh A liver function, normal serum bilirubin without clinically significant portal hypertension. And it was expected to be able to give a chance for better long-term OS and DFS (21). A meta-analysis and review indicated that portal hypertension had an adverse impact on short- and long-term outcomes for HCC patients undergoing partial hepatectomy (22). Meanwhile, liver cirrhosis was considered as one of the most important risk factors for surgical outcomes in HCC patients (3, 16, 23, 24). In the present study, the 5-year OS and DFS in HCC patients with liver cirrhosis are 63.2 and 38.1%, whereas 82.7 and 59.8% for those without liver cirrhosis. Furthermore, the results suggested that surgical outcomes decreased significantly with the increasing degrees of liver cirrhosis. A study from Kim et al. (9) showed that histological sub-classification of liver cirrhosis according to the Laennec scoring system was a significant predictor of late recurrence in HBV-induced HCC patients after surgical resection. However, due to the small study population and short follow-up period, the results might be unconvincing. Our subgroup analysis showed that the 5-year OS and DFS were 54.7 and 28.8% in HCC patients with moderate or severe liver cirrhosis, which was significantly better than the results in our previous study (45 and 25%) (17). This was probably because of the fact that we excluded the cases with clinical significant portal hypertension in this study. Accordingly, the surgical outcomes were relatively good. Our results suggested that the severity of cirrhosis was inversely correlated with surgical prognosis of HCC patients even in those HCC patients without clinical portal hypertension. Moreover, cox-regression analysis suggested that F4B/4C stage was an independent risk factor for tumor recurrence and long-term outcomes after surgical resection. The mechanism of tumor recurrences which are prone to occurring in cirrhotic liver remains unclear. The poor surgical outcomes associated with liver cirrhosis have been hypothesized by previous studies. Hepatitis-induced repeated inflammation and cellular necrosis might led to hepatocyte proliferation and increase random gene mutations, which would accelerate the carcinogenesis of HCC (25, 26).

Perioperative mortality was not observed in the present study. Major complications were significantly higher for patients in the F4B/F4C group than those in the no/F4A group (17.0% vs 7.4%, *p <*0.001). The results seemed to be consistent with the aforementioned confirmed correlation among histological sub-classification of cirrhosis and clinical stages of portal hypertension which could reflect reserve liver function (15). HCC recurrence was found in more than half of patients who underwent curative surgical resection during the follow-up period. Subgroup analysis indicated that tumor recurrence was more frequent in the F4B/4C group than in the no/F4A group. Tumor recurrence over 2 years was believed to be *de novo* new tumor and coexistence of moderate or severe liver cirrhosis were more likely relapse (27). Theoretically, anatomic resection was effective for eradication of the intrahepatic metastases of HCC though portal vein system and thus decreased tumor recurrence (28). However, most HCC patients with moderate or severe liver cirrhosis were not suitable for major anatomic resection owing to their impaired hepatic reserve function. Only thirty-six HCC patients (21.7%) underwent anatomic LR and mostly performed in patients with no or mild liver cirrhosis. We also found that late recurrence was more frequent in the F4B/4C group than those in the no/F4A group (66.3% vs 23.5%, *p <*0.001), which was consistent with the results of previous studies (16, 27).

Some studies illustrated that tumor size was one of the most important risk factors for surgical outcomes in HCC patients for the reason that increasing tumor size was associated with the presence of microscopic vascular invasion (MVI) (4, 21). However, in the present study, the results showed that tumor size was not associated with worse prognosis in HCC patients with lesions ≤5 cm, which was inconsistent with the results from the previous studies. The most plausible explanation for this inconsistence was the small sample size and HCC patients with portal hypertension were excluded in our study.

It is widely known that MVI and absence of tumor capsule are strongly related to survival and recurrence after LR for HCC patients (4, 6, 29). Previous studies reported that the rates of MVI incidence ranged from 15 to 33% (16, 30–32) in excised HCC which is higher than our current finding of 10.8%. Nagano et al. (33) demonstrated that tumors larger than 7 cm, single nodular type tumors without regular tumor capsule were associated with an increased risk of MVI. Small tumor size in the present study may be responsible for the relative lower MVI incidence. Our findings showed that MVI was an important risk factor for predicting the long-term outcomes of HCC patients who underwent LR, which was consistent with previous study (4). Sumie et al. (34) recognized the impact of MVI on surgical outcomes and sought for a model to predict patients who were at an increased risk for having MVI. They hoped that its ability to predict the likelihood of MVI would be helpful to decide the appropriate treatment modalities. The underlying mechanism of MVI and absence of tumor capsule adversely affecting surgical outcomes remain unclear. A study suggested that the portal vein acted as an efferent vessel for tumor cells and it also was the path for tumor cell infiltration or expansion. Tumor cells invaded efferent vessels and then extended beyond the capsule to the portal vein branches (35). However, the number of patients with MVI in this study was only eighteen patients and fifty-four patients in absence of tumor capsule. Multi-center studies are necessary to establish the confirmative role of MVI and absence of tumor capsule for HCC recurrence after LR.

There are still several limitations in our study. At first, small study population of HCC patients and retrospective study might increase selection bias and weaken the statistical strength. Furthermore, because preoperative hepatic venous pressure gradient (HVPG) was not routinely measured in our center before April 2018, portal hypertension was indirectly defined as presence of esophageal varices or a platelet count <100,000/ml combined with splenomegaly. The accuracy decreased accordingly. Thirdly, routine antiviral treatment for HBV after LR may slow down the progression of liver cirrhosis, and the status of cirrhotic severity after LR during the follow-up period could not be accurately assessed due to the fact that liver biopsies could not be performed repeatedly in clinical settings. Multi-center studies with large sample should be carried out to provide stronger evidence to get more convincing results. Fourthly, although the present study showed the clinical importance of cirrhotic severity, it is of no use to choose suitable candidates for LR or predict surgical complications due to histological evaluation obtained after LR. Thus, preoperative prediction of severity of cirrhosis using non-invasive methods is urgent to be investigated. Finally, MVI-positive was reported to be an important risk of intrahepatic metastatic after curative liver resection, therefore, systemic therapy might improve recurrence and long-term outcomes for this subgroup of HCC patients (36). However, eighteen HCC with MVI-positive did not receive systemic therapy after surgical resection at that time.

## Conclusion

In summary, results from the present study emphasized the importance of histological severity of liver cirrhosis in surgical outcomes for HCC patients. Microscopic vascular invasion, absence of tumor capsule and severity of liver cirrhosis were independent risk factors of surgical outcomes for early HCC patients without portal hypertension. Histological severity of liver cirrhosis affected surgical outcomes of early-stage HCC patients independently of portal hypertension and gave useful prognostic information that aided in the optimal management of early-stage HCC patients in absence of portal hypertension.

## Data Availability Statement

The original contributions presented in the study are included in the article/**Supplementary Material**. Further inquiries can be directed to the corresponding author.

## Ethics Statement

This study was approved by the Medical Ethics Committee of Tongji Hospital, Huazhong University of Science and Technology, Wuhan, China. The patients/participants provided their written informed consent to participate in this study.

## Author Contributions

E-lZ and Z-yH made the concept and design. JiangL, JianL, W-qW, and JG acquired and analyzed the data. E-lZ drafted the manuscript. Z-yH critically revised the manuscript for the final version. E-lZ and JiangL conducted the statistical analysis. All authors contributed to the article and approved the submitted version.

## Funding

This work was supported by the grants from National Natural Science Foundation of China (81902839) and National Science and Technology Major Project of China (2017ZX10203207-002).

## Conflict of Interest

The authors declare that the research was conducted in the absence of any commercial or financial relationships that could be construed as a potential conflict of interest.

## Supplementary Material

Supplementary Figure 1 The flow chart of the present study.

Supplementary Figure 2 Overall survival **(A)** and disease-free survival **(B)** outcomes of HCC patients with different degrees of liver cirrhosis according to the Laennec scoring system (*P *< 0.001).
