# Supplementary figures and images for: Sub-Classification of Cirrhosis Affects Surgical Outcomes for Early Hepatocellular Carcinoma Independent of Portal Hypertension
Source: Front Oncol. 2021 May 20;11:671313. doi: 10.3389/fonc.2021.671313 (PMC8173036; doi:10.3389/fonc.2021.671313)

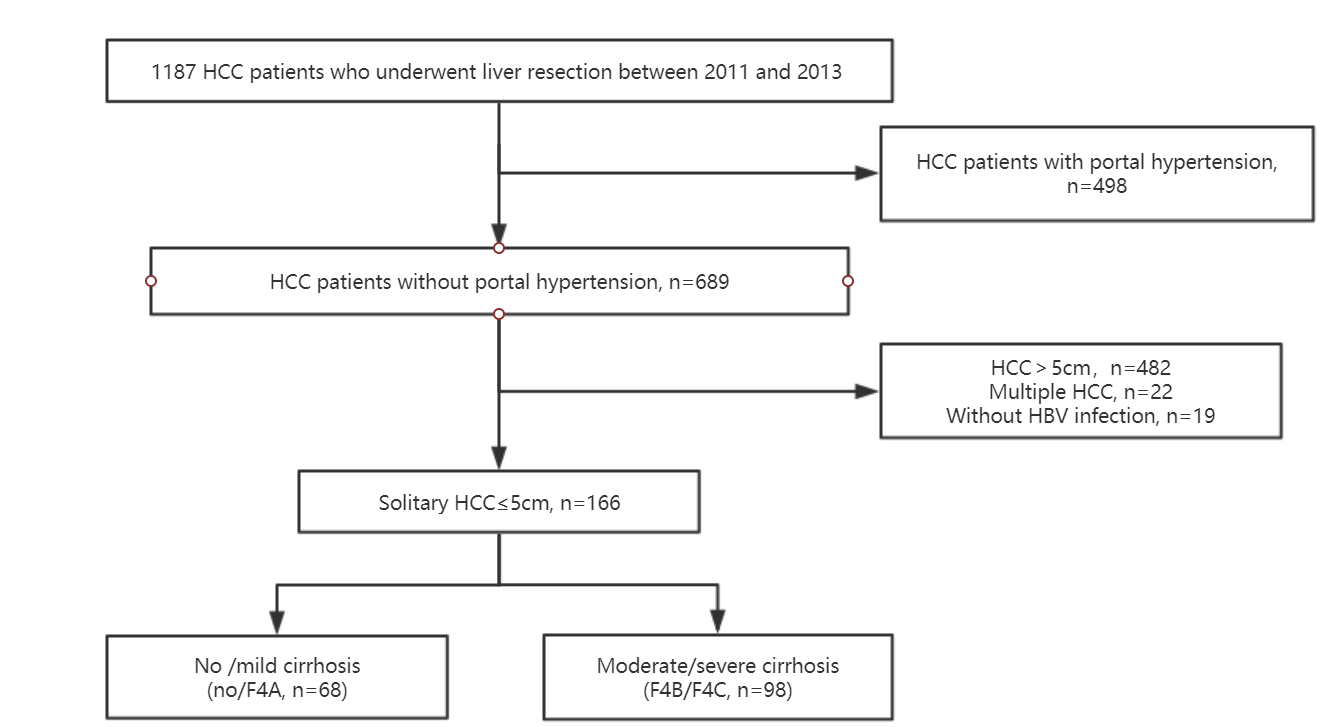

Supplement: Supplementary Figure 1 — The flow chart of the present study. [file Image_1.tif]

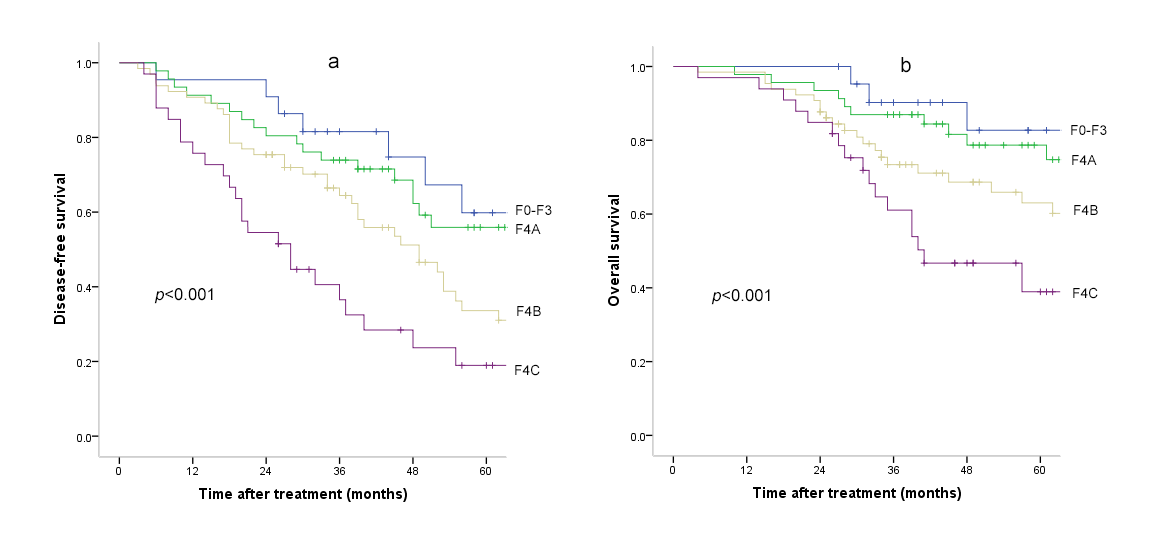

Supplement: Supplementary Figure 2 — Overall survival (A) and disease-free survival (B) outcomes of HCC patients with different degrees of liver cirrhosis according to the Laennec scoring system (P < 0.001). [file Image_2.tif]
