# Supplementary material for: Sub-Classification of Cirrhosis Affects Surgical Outcomes for Early Hepatocellular Carcinoma Independent of Portal Hypertension
Source: Front Oncol. 2021 May 20;11:671313. doi: 10.3389/fonc.2021.671313 (PMC8173036; doi:10.3389/fonc.2021.671313)
Supplement: Supplementary file 3 [file Table_1.docx]

**Original research**

**Short running title:** Outcomes for HCC patients without portal hypertension

**Sub-classification of Cirrhosis Affects Surgical Outcomes for Early Hepatocellular Carcinoma independent of Portal Hypertension**

Er-lei Zhang, M.D., Jiang Li, M.D., M.D., Jian Li, M.D., Wen-qiang Wang, M.D., Jin Gu, M.D., Zhi-yong Huang, M.D.*

Hepatic Surgery Center, Tongji Hospital, Tongji Medical College

Huazhong University of Science and Technology, Wuhan, China, 430030

＊Please address correspondence to: Dr. Zhi-yong Huang, M.D.

Professor of Surgery, Hepatic Surgical Center,

Tongji Hospital, Tongji Medical College

Huazhong University of Science and Technology

1095 Jie Fang Da Dao, Wuhan, China, 430030

Tel: +86-27-83665392

Fax: +86-27-83663432

E-mail: huangzy@tjh.tjmu.edu.cn

**Table 1.** Perioperative details of HCC patients in the no/F4A versus F4B/F4C group.

|  | No/F4A (n=68) | F4B/4C (n=98) | *p*-value |
| --- | --- | --- | --- |
| Blood loss, mL | 214.5±124.0 | 307.7±252.8 | 0.008 |
| Blood transfusion, n (%) | 2(2.9) | 13(13.3) | 0.023 |
| Pringle maneuver, n (%) | 11(16.2) | 32(32.7) | 0.017 |
| Operative time, min (±SD) | 194.2 (±102.06) | 224.2 (±125.02) | 0.159 |
| *Hospital stay, days (±SD) | 8±3.6 | 12±5.8 | <0.001 |
| Major complication | 5 | 15 | <0.001 |
| pleural effusion | 3 | 7 |  |
| significant ascites | 1 | 4 |  |
| bile leakage | 1 | 3 |  |
| liver failure | 0 | 1 |  |

*Hospital stay mean days from operation to hospital discharge.

**Table 2.** Baseline patient characteristics in the no/F4A versus F4B/F4C group.

|  | No/F4A (n=68) | F4B/4C (n=98) | *p*-value |
| --- | --- | --- | --- |
| Age (years) | 49.3±10.6 | 47.6±9.9 | 0.311 |
| Sex (Male:Female) | 62:6 | 86:12 | 0.614 |
| ALT (U/L) | 35.0±18.8 | 39.8±19.5 | 0.123 |
| AST (U/L) | 31.5±12.6 | 33.9±14.2 | 0.296 |
| Total bilirubin ( umol/L) | 12.6±3.3 | 13.4±4.2 | 0.175 |
| AFP (ng/ml) |  |  | 0.812 |
| >400 (n, %) | 22(32.4) | 30(30.6) |  |
| ≤400 (n, %) | 46(67.6) | 68(69.4) |  |
| Platelet count (x10^9^/L) | 129.0±47.0 | 124.2±50.6 | 0.705 |
| Spleen thickness (cm) | 3.8±0.6 | 4.0±0.7 | 0.241 |
| ICGR-15 (%) | 4.3±2.6 | 5.4±3.3 | 0.129 |
| Tumor size (cm) | 4.0±0.9 | 3.8±1.0 | 0.240 |
| Tumor differentiation (n, %) |  |  | 0.229 |
| Well | 18(26.5) | 22(22.4) |  |
| Moderate | 39(57.4) | 49(50.0) |  |
| Poor | 11(16.1) | 27(27.6) |  |
| Microscopic vascular invasion (n,%) | 7(10.3) | 11(11.2) | 0.850 |
| Capsule (n, %) | 46(67.6) | 66(67.3) | 0.751 |
| ASA score I/II | 32/36 | 47/51 | 0.909 |
| BMI | 23.3±3.1 | 23.2±2.9 | 0.845 |
| Type of resection (n, %) |  |  | <0.001 |
| Right anterior sectionectomy | 7(10.3) | 1(1.0) |  |
| Right posterior sectionectomy | 3(4.4) | 1(1.0) |  |
| Left hepatectomy | 4(5.9) | 3(3.1) |  |
| Left lateral sectionectomy | 11(16.1) | 6(6.1) |  |
| Local resection | 43(63.2) | 87(88.8) |  |

**Table 3.** Treatment of HCC recurrences in this study

|  | Treatment | | | | |
| --- | --- | --- | --- | --- | --- |
| Recurrence(n) | LR | MCT | TACE | MCT+TACE | TACE+  chemotherapy |
| Overall(88),n(%) | 10 (11.4%) | 38(43.3%) | 24(27.3%) | 10(11.4%) | 6(6.8%) |
| No/Mild(27),n(%) | 7 (25.9%) | 12(44.4%) | 4(14.9%) | 2(7.4%) | 2(7.4%) |
| Moderate/severe(61),n(%) | 3 (4.9%) | 26(42.6%) | 20(32.8%) | 8(13.1%) | 4(6.6%) |

**Table 4.** Overall survival (OS) and Disease-free survival (DFS) in HCC patients with different degrees of liver cirrhosis.

|  | 1-year(%) | 3-year(%) | 5-year(%) | *p-* value |
| --- | --- | --- | --- | --- |
| Overall survival |  |  |  | <0.001 |
| F0-F3 (22) | 100 | 90.2 | 82.7 |  |
| F4A (46) | 97.8 | 87.0 | 78.7 |  |
| F4B (65) | 98.5 | 73.4 | 63.0 |  |
| F4C(33) | 97 | 61.6 | 38.9 |  |
| Disease-free survival |  |  |  | <0.001 |
| F0-F3 (22) | 95.5 | 81.6 | 59.8 |  |
| F4A (46) | 91.3 | 73.9 | 55.9 |  |
| F4B (65) | 90.8 | 64.5 | 33.6 |  |
| F4C(33) | 75.8 | 36.5 | 18.9 |  |
